# Extracts, Fractions, and Subfractions from Purple-Orange Sweet Potato (*Ipomoea batatas* L.): Xanthine Oxidase Inhibitory Potential and Antioxidant Properties

**DOI:** 10.3390/molecules30112442

**Published:** 2025-06-03

**Authors:** Hendy Suhendy, Muhamad Insanu, Irda Fidrianny

**Affiliations:** 1 Department of Pharmaceutical Biology, School of Pharmacy, Bandung Institute of Technology, Bandung 40132, Indonesia; 30722301@mahasiswa.itb.ac.id (H.S.); irda@itb.ac.id (I.F.); 2 Department of Biology Pharmacy, Faculty of Pharmacy, University of Bakti Tunas Husada, Tasikmalaya 46115, Indonesia

**Keywords:** purple-orange sweet potatoes, extracts, fractions, subfractions, xanthine oxidase inhibitory, antioxidant

## Abstract

Previous research has shown that fractions outperformed extracts in pharmacological activity and safety. This study assessed the total phenol and flavonoid content, as well as antioxidant and xanthine oxidase inhibitory (XOI) activities, of purple-orange sweet potato extracts, fractions, and subfractions. Using UV-visible spectrophotometry, the leaves showed the highest values for total phenol, flavonoid, 2,2-diphenyl-1-picrylhydrazyl (DPPH), Ferric Reducing Antioxidant Power (FRAP), Cupric Ion Reducing Antioxidant Capacity (CUPRAC) assays, and XOI activity. The sequential extraction of the leaves yielded ethyl acetate extract as the most potent, with a yield of 10.4%, a DPPH assay result of 511.212 ± 0.416 mg ascorbic acid equivalent antioxidant capacity (AEAC)/g, and XOI activity of 45.192 ± 4.981 mg allopurinol equivalent xanthine inhibitory capacity (AEXIC)/g. CF5 had the greatest DPPH assay (158.475 ± 0.170 mg AEAC/g), FRAP assay (86.849 ± 0.048 mg AEAC/g), CUPRAC assay (1008.892 ± 1.620 mg AEAC/g), and XOI activity (6.062 ± 1.730 mg AEXIC/g) values. Subfraction CSF3 from fraction CF5 was analyzed using UPLC-MS/MS and revealed the presence of compounds such as cholest-4-en-3-one, 4-hydroxy-6-[2-(2-methyl-1,2,4a,5,6,7,8,8a-octahydronaphthalen-1-yl) ethyl] oxan-2-one, tangeritin, 4-methyl benzophenone, benzophenone, (+)-ar-turmerone, 4-methoxycinnamic acid, and ricinine. This study was the first to report xanthine oxidase inhibitory activity in allopurinol equivalence. The leaves of the purple-orange sweet potato showed potential as a source of natural antioxidants.

## 1. Introduction

An antioxidant is a molecule that can inhibit the oxidation of other molecules. It disrupts the free radical chain of reactions by donating its electrons to stabilize free radicals without becoming free radicals themselves [1]. In certain situations, the body’s defense system (antioxidant enzymes and compounds like glutathione, vitamins, and coenzyme Q) needs external support to counteract excess free radicals. This support comes from dietary antioxidants in fruits, vegetables, and other plants [2]. Sweet potato (*Ipomoea batatas* L.) contains antioxidant compounds, and various parts of the plant are known to exhibit antioxidant activity. Sweet potato has advantages over other plants in terms of abundance and resilience. It is a key food crop globally, ranking sixth in importance worldwide and eighth in the tropics, with Indonesia being one of the largest producers [3]. Sweet potato also tolerates various conditions such as drought, heat stress, salinity, and ozone exposure [4]. Therefore, sweet potato is a suitable raw material for the development of natural antioxidant products.

Previous research has shown that extracts from the tuber part of the sweet potato have strong antioxidant activity [5]. Other studies have found that extracts from the leaves exhibit strong antioxidant activity [6,7]. Tuber extracts from the sweet potato purple-orange variety had the best antioxidant potential compared to other varieties [8]. Compounds such as anthocyanins, chlorogenic acid, ferulic acid, and β-carotene, which contribute to antioxidant activity, are present in sweet potatoes, including this variety [9].

Xanthine oxidase generates oxygen-free radicals, especially during reperfusion, which produce superoxide radicals. Flavonoids like quercetin and silibin inhibit its activity, reducing oxidative damage, with luteolin identified as the most potent inhibitor [10,11,12,13]. Both in silico and in vivo studies have demonstrated that extracts and fractions from sweet potato inhibited xanthine oxidase activity [14,15].

Current research on natural products, particularly medicinal plants, is increasingly emphasizing fraction exploration due to the higher pharmacological activity typically found in these forms compared to their extracts [16,17,18,19,20,21,22,23]. Other research has indicated that fractions have a good safety profile [24,25,26]. The benefits of fractions in terms of efficacy and safety may explain their preference for various herbal products. Examples include ginseng fractions used as immunomodulators and fractions derived from *Anoectochilus formosanus* for chemoprevention or treatment of human malignancies [27,28]. Considering this trend, further exploration at the subfraction level is justified to confirm their pharmacological activities and safety profiles.

Research on sweet potato plants has primarily focused on the extracts and fractions from their leaves and tubers [5,6,7,8,14,15]. Therefore, it is understandable that sweet potato has not yet been widely developed into herbal products. Furthermore, there are no reports on the antioxidant and xanthine oxidase inhibitory (XOI) potential of sweet potato subfractions, particularly from the purple-orange variety; thus, bioassay-guided subfractionation is necessary. This study investigates the total phenolic and flavonoid contents, antioxidant activities, and xanthine oxidase inhibitory activities from three parts (leaves, stems, and tubers) of purple-orange sweet potato plants, as well as extracts from selected plant parts, fractions from selected extracts, and subfractions from selected fractions, along with the tentative identification of active compounds in the selected subfractions. In addition, this study assesses the potential of the purple-orange sweet potato variety to be developed into a herbal product, whether as an extract, fraction, or subfraction.

## 2. Results

### 2.1. Bioactive Content, Inhibitory of Xanthine Oxidase and Antioxidant Activities of Ethanolic Extracts from Three Plant Parts

The total phenolic and flavonoid content, xanthine oxidase inhibitory (XOI) activity, and antioxidant activity were tested on ethanol extracts from the stems, tubers, and leaves of purple-orange sweet potato plants (ESE, ETE, and ELE). The data on total phenolic and flavonoid content, antioxidant (DPPH, FRAP, CUPRAC) activity, and XOI activity among the ethanol extracts of the three plant parts showed significant differences (*p* < 0.05). In general, in many publications, the xanthine oxidase inhibitory activity was expressed in percentage inhibitory activity at a specific concentration or IC_50_, the concentration of the sample that can inhibit 50% of enzyme activity. In this research, the XOI activity for the first time was presented as mg allopurinol equivalent xanthine oxidase inhibitory capacity (AEXIC) per g sample. This means that the xanthine oxidase inhibitory capacity of a 1 g sample is similar to that of a certain mg of allopurinol. Table 1 shows that the highest yield was from the leaves, while the lowest was from the tubers. Meanwhile, ELE exhibited the highest values in each test.

### 2.2. Thin Layer Chromatography, Bioactive Content, Xanthine Oxidase Inhibitory Activity, and Antioxidant Activity of Leaf Extracts

**Figure 1:**
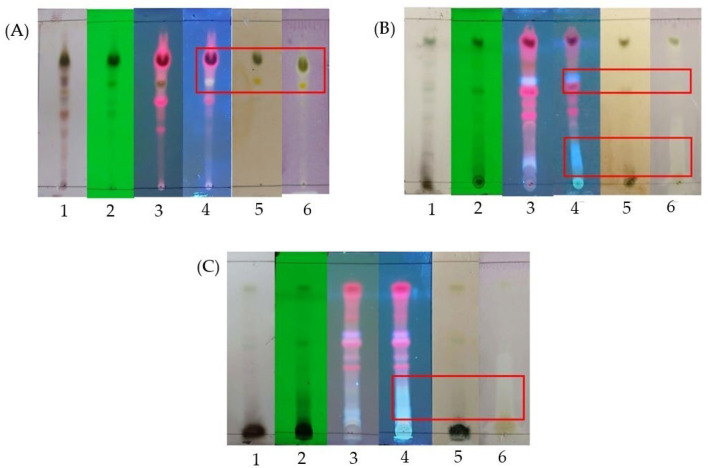
TLC monitoring of purple-orange sweet potato leaves extracts; silica gel 60 GF254 as stationary phase. (**A**) n-hexane extract, mobile phase: n-hexane–chloroform (1:9); (**B**) ethyl acetate extract, mobile phase: chloroform–ethyl acetate (7:3); (**C**) ethanol extract, mobile phase: chloroform–methanol–water (8:2:1): 1 = sprayed with 5% H_2_SO_4_; 2 = under UV λ 254 nm; 3 = under UV λ 366 nm; 4 = sprayed with citroborate, under UV light λ 366 nm; 5 = sprayed with 10% FeCl_3_; 6 = sprayed with 0.2% DPPH. 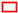 = there is a qualitative correlation between phenolic and flavonoid compounds with antioxidant activity.

Thin layer chromatography (TLC), total phenolic and flavonoid content, antioxidant activity, and XOI activity were tested on n-hexane, ethyl acetate, and ethanolic extracts (HE, EAE, and EE) from the leaves of Ipomoea batatas plants. Thin layer chromatography (TLC) was performed to identify the profiles of secondary metabolites and antioxidant activities in the three extracts. The TLC results in Figure 1 show the presence of phenolic compounds, flavonoids, and antioxidant activity. Table 2 illustrates that the ethyl acetate extract yield was the highest among all the extracts, indicating that the leaves of the purple-orange sweet potato contain more semi-polar compounds. Meanwhile, the data on total phenolic and flavonoid content, antioxidant (DPPH, FRAP, CUPRAC), and XOI activities among the three extracts of leaves (HE, EAE, and EE) showed significant differences (*p* < 0.05). EE had the highest total phenolic content, while HE contained the greatest number of flavonoids. EE demonstrated the strongest antioxidant activity in the DPPH, FRAP, and CUPRAC assays, whereas EAE exhibited the highest XOI activity. Meanwhile, the correlations between phenolic and flavonoid content to antioxidant activity and XOI are exposed in Table 3.

### 2.3. Thin Layer Chromatography, Bioactive Content, Xanthine Oxidase Inhibitory and Antioxidant Activities of Fractions

**Figure 2:**
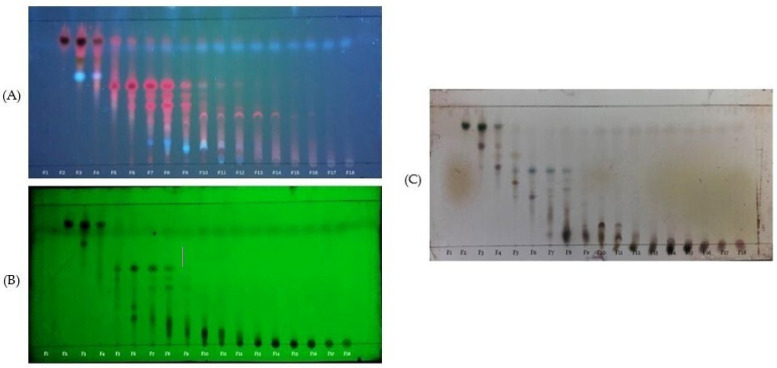
TLC monitoring of fraction from the ethyl acetate leaves extract; stationary phase silica gel 60 GF254; mobile phase: chloroform–ethyl acetate (7:3). (**A**) under UV λ 366 nm; (**B**) under UV λ 254 nm; (**C**) sprayed with 5% H_2_SO_4_. F1-F18 = fractions 1–18.

**Figure 3:**
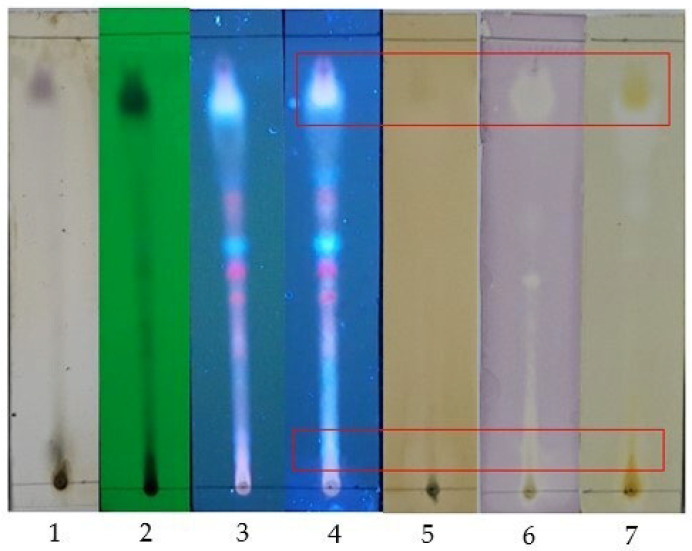
TLC monitoring of CF5; stationary phase silica gel 60 GF254; mobile phase: chloroform–ethyl acetate (7:3); 1 = sprayed with 5% H_2_SO_4_; 2 = under UV λ 254 nm; 3 = under UV λ 366 nm; 4 = sprayed with citroborate, under UV λ 366 nm; 5 = sprayed with 10% FeCl_3_; 6 = sprayed with 0.2% DPPH; 7 = sprayed with CUPRAC; 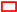 = there is a qualitative correlation between phenolic and flavonoid compounds with antioxidant activity.

The ethyl acetate leaves extract (EAE) was fractionated by vacuum liquid chromatography, producing 18 fractions. Then, 18 fractions were monitored by TLC. Based on similar TLC profiles, five combined fractions were obtained (Figure 2): fractions 2–4 (CF1), fractions 5–6 (CF2), fractions 7–9 (CF3), fractions 10–12 (CF4), and fractions 13–18 (CF5). The polarity levels of the compounds in the combined fractions were as follows: CF1 < CF2 < CF3 < CF4 < CF5. As shown in Table 4, CF3 had the highest yield, while CF2 had the lowest. All test data revealed significant differences among the five combined fractions except for XOI activity (*p* < 0.05). The greatest total flavonoid and phenolic content was found in CF3, the highest antioxidant activity (DPPH, FRAP, CUPRAC) was observed in CF5, and the highest xanthine oxidase inhibition was seen in CF2 and CF5. Figure 3 illustrates a qualitative correlation between the phenolic and flavonoid compounds in CF5 and its DPPH and CUPRAC antioxidant activities.

### 2.4. Bioactive Content, Xanthine Oxidase Inhibitory and Antioxidant Activities of Subfractions

**Figure 4:**
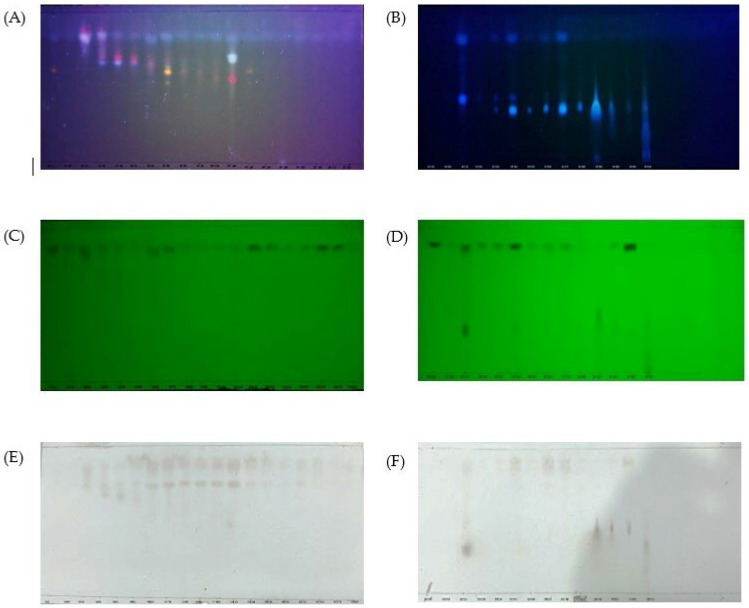
TLC monitoring of subfraction from CF5; stationary phase silica gel 60 GF254; mobile phase: chloroform–ethyl acetate (7:3). (**A**,**B**) = under UV λ 366 nm; (**C**,**D**) = under UV λ 254 nm; (**E**,**F**) = sprayed with 5% H_2_SO_4_. SF1-SF305 = subfractions 1–305.

CF5 was subfractionated by classic column chromatography, and 305 subfractions were obtained. Then, subfractions were monitored by TLC. Based on similar TLC profiles, five combined subfractions were obtained (Figure 4): subfractions 1–56 (CSF1), subfractions 57–126 (CSF2), subfractions 127–204 (CSF3), subfractions 205–286 (CSF4), and subfractions 287–305 (CSF5). The polarity levels of the compounds in the combined subfractions were as follows: CSF1< CSF2< CSF3< CSF4< CSF5.

The highest yield was observed in CSF3, while the lowest was in CSF5 (Table 5). The total phenolic and flavonoid content, inhibitory activity of xanthine oxidase, and antioxidant activity were assessed for the combined subfractions. All test data showed significant differences (*p* < 0.05) among the five combined subfractions, except for the DPPH assay between CSF2 and CSF5, as well as CSF3 and CSF4. The greatest total flavonoid and phenolic content was found in CSF2, the highest antioxidant activity (DPPH) was observed in CSF1, and the highest xanthine oxidase inhibitory activity was seen in CSF4. When evaluated using the combined scores for yield, TFC, TPC, antioxidant activity, and xanthine oxidase inhibitory activity, CSF3 showed the most promising potential, and the compound in CSF3 was further analyzed.

### 2.5. Comparison of Xanthine Oxidase Inhibitory and Antioxidant Activities of ELE, EAE, CF5, and CSF3

The xanthine oxidase inhibitory and antioxidant activities data for ELE, EAE, CF5, and CSF3 were statistically analyzed to determine significant differences between the groups. Table 6 revealed significant differences among the groups for each test (*p* < 0.05). Generally, the leaf fractions expressed lower antioxidant and XOI activities than their extracts, except in the CUPRAC method.

### 2.6. Analysis of Secondary Metabolites in CSF3 Using UPLC–MS/MS

**Figure 5:**
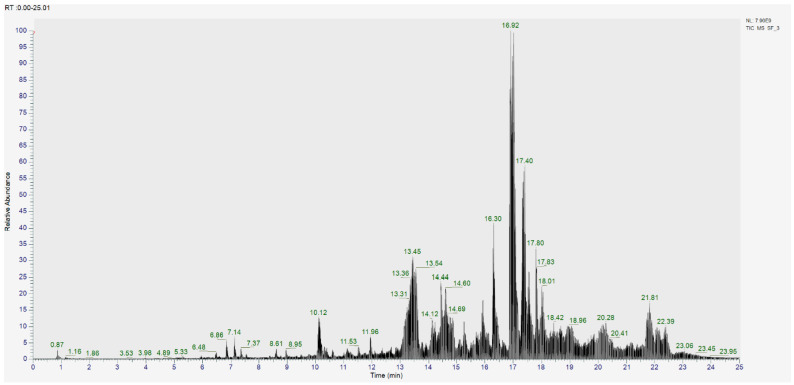
UPLC–MS/MS chromatogram of the sample analyzed under the following conditions: temperature set at 40 °C, flow rate of 0.3 mL/min, sample concentration of 1 mg/mL in 70% methanol (MeOH), and an injection volume of 3 µL.

The analysis of CSF3 from CF5 using UPLC–MS/MS (Figure 5 and Table 7) revealed the presence of compounds such as cholest-4-en-3-one (0.11%), 4-hydroxy-6-[2-(2-methyl-1,2,4a,5,6,7,8,8a-octahydronaphthalen-1-yl)ethyl]oxan-2-one (0.02%), tangeritin (0.01%), 4-methyl benzophenone (0.01%), benzophenone (0.06%), (+)-ar-turmerone (0.02%), 4-methoxy cinnamic acid (0.5%), and ricinine (0.02%).

## 3. Discussion

The total phenols and flavonoids data in Table 1 were obtained using 96% ethanol as the solvent. Previous studies have demonstrated that the ethanol–water ratio influences total phenolic content in extracts [7]; therefore, these values may vary if a different ethanol–water solvent ratio is used. The table demonstrates that the highest yield comes from the leaves, consistent with previous research reporting that the leaves produced the highest ethanol extract among various plant parts [29,30]. This is because plants use leaves besides their chlorophyll content for various metabolic processes that produce high concentrations of secondary metabolites and other bioactive compounds [31]. The other studies also confirmed that among different plant parts, the tubers have the lowest yield [32,33]. The leaves’ high phenolic and flavonoid content (Table 1) is due to their role as the center of photosynthesis, leading to greater production of these compounds compared to other plant parts. These secondary metabolites contribute to antioxidant and XOI activities. The higher the total flavonoid content, the greater the antioxidant activity [8]. Research on *Amaranthus cruentus* and *Choerospondias axillaris* has shown a strong correlation between xanthine oxidase (XO) inhibitory activity and the total phenolic and flavonoid content [34,35]. The leaves are now considered a promising source for natural antioxidant products and were selected for further extraction based on their yield, total flavonoid and phenolic content, xanthine oxidase inhibition, and antioxidant activity.

Figure 1 demonstrates that the TLC results were consistent with previous research, which established a qualitative correlation between phenolic and flavonoid compounds in n-hexane, ethyl acetate, and ethanol extracts and their antioxidant activities [8]. Nevertheless, quantitatively, only the total phenolic content in the leaf’s extracts exhibited a perfect correlation with antioxidant activity, particularly in the DPPH and FRAP assays (Table 3). Meanwhile, Table 2 indicates that the leaves contained a higher amount of semi-polar compounds, supporting earlier findings that leaf extracts obtained using ethyl acetate yield more than the other solvents [36,37]. The highest total phenolic content in EE and the greatest flavonoid content in HE aligned with previous studies, which reported that ethanol leaf extracts of purple sweet potato tubers contained the highest total phenolics, with values of 19.64 g GAE/100 g [6]. Additionally, the highest total flavonoid content in the purple-orange variety of sweet potato was found in HE, with a value of 12.79 g QE/100 g [8]. The high flavonoid content in the n-hexane extract may be attributed to the occurrence of methylated flavonoids that react with AlCl_3_ [38]. EE showed the highest antioxidant activity (DPPH, FRAP, CUPRAC), whereas EAE exhibited the highest XOI activity. These results aligned with earlier research that indicated that the ethanol extract of sweet potato leaves from red-purple tuber and purple tuber provided the highest antioxidant activity compared to the other extracts, with percentage values of 57.53% and 64.85% for the ABTS assay and 97.63% and 93.34% for the DPPH assay [6]. Meanwhile, the other research denoted that the ethyl acetate extract of *Cratoxylum glaucum* leaves exhibited the highest XOI activity compared to other extracts, with an IC_50_ of 56.16 µg/mL [39]. Antioxidant and XOI activities are not solely influenced by high total phenolic and flavonoid contents but also by the structure and potential redox of these secondary metabolites [40]. For example, flavonoids with a C2=C3 double bond and hydroxyl groups at C-7 and C-5 enhance xanthine oxidase inhibition, while hydroxyl groups at C3 and C6 reduce this activity [41]. DPPH antioxidant activity is higher when flavonoids have OH groups at positions C3′, C4′, C3, a double bond at C2 and C3, and a keto group at C4. Flavonoids with O-methylation or O-glycosylation will reduce the antioxidant capacity. Methylation at C4′ and C6′ eliminates the ability to neutralize DPPH [42,43,44,45,46]. Based on the yield and XOI activity, EAE was further fractionated.

Figure 3 revealed that the phenolic and flavonoid compounds in CF5 have a qualitative correlation with DPPH and CUPRAC antioxidant activities. The presence and concentration of other compounds, such as terpenes and alkaloids, might affect these activities [47,48]. The high antioxidant activity of CF5, as observed in previous studies [49], is likely due to the presence of more polar compounds than other combined fractions. The polarity of flavonoid compounds depends on hydroxyl groups; the more hydroxyl groups there are, the more polar the compound. As previously explained, the existence of hydroxyl groups at the C3′, C4′, and C3 positions of flavonoid compounds enhances DPPH antioxidant activity, while hydroxyl groups at C5 and C7 can increase XOI activity [41,42,43,45,46]. Based on its antioxidant and XOI activities, CF5 was selected for further subfractionation. CF5 was the most promising candidate for developing traditional medicine and warrants further research to identify its active compounds.

The highest antioxidant and xanthine oxidase inhibitory activities were not expressed by the greatest total phenolic or flavonoid content in the CSF, indicating that these compounds may not have been the primary contributors to the observed activities (Table 5).

Table 6 demonstrated a decreasing trend from the universal solvent extract (ELE) to the semi-polar solvent extract (EAE), and further to the fraction (CF5). Synergistic constituent interactions in plant extracts may decrease activity when the compounds are isolated [50]. The previous studies have reported similar findings when comparing the pharmacological activity of extracts with their fractions. The DPPH values for the methanolic extract, n-butanol fraction, chloroform fraction, ethyl acetate fraction, and n-hexane fraction of *Cichorium intybus* seeds had IC_50_ values of 21.28, 38.25, 29.42, 50.21, and 72.14 μg/mL, respectively. A study on *Polygonum hydropiper* expressed that the crude methanol extract had an IC_50_ of 29.27 μg/mL for XOI activity, which was much lower (indicating stronger activity) than the ethyl acetate fraction’s IC_50_ of 165.25 μg/mL [51,52]. The DPPH assay values for CSF3 were lower than CF5, indicating that the antioxidant activity of the subfraction was weaker than that of the parent fraction. This aligned with previous findings, where DPPH radical-scavenging activity in *S. latiuscula* subfractions ranged from 90.23% to 93.65%, compared to 94.08% for the parent fraction at 50 µg/mL [53]. In contrast, the XOI activity of CSF3 was higher than that of CF5, demonstrating better activity in this subfraction. This is consistent with earlier research where the EC_50_ values of *Carissa opaca* root subfractions ranged from 8.40 to 11.66 µg/mL, compared to 129 µg/mL for the ethyl acetate fraction [54].

Table 7 denotes the presence of some secondary metabolites. Cholest-4-en-3-one (4-cholestenone, 4-STN) is a steroid characterized by a 3-oxo functional group at the carbon-3 position and a double bond between carbon-4 and carbon-5 in its structure [55]. No reports confirm that this compound exhibits antioxidant and XOI activities. However, previous research has shown that some steroid compounds possess both activities [56,57]. One compound tentatively predicted from CSF3 was 4-hydroxy-6-[2-(2-methyl-1,2,4a,5,6,7,8,8a-octahydronaphthalen-1-yl) ethyl] oxan-2-one, classified as a delta-lactone [58]. This compound had strong antioxidant activity, with IC_50_ values ranging from 1.2 to 5.4 μg/mL [59]. Although its XOI activity was unknown, related compounds like gamma-lactones have shown promising activity, with IC_50_ values of 79.0 μg/mL [60]. Tangeritin demonstrated 93% DPPH scavenging antioxidant activity at a concentration of 67.5 µg/mL, while its XOI activity in propolis varies, with IC_50_ values ranging from 0.4 to 0.8 μg/mL [61,62]. No prior reports have been found regarding 4-methyl benzophenone. However, like other benzophenone derivatives, this compound likely possesses potential antioxidant and XOI activities. Benzophenone compounds such as annulatophenonoside, acetylannulatophenonoside, and annulatophenone exhibited antioxidant activity, with ABTS scavenging activity exceeding 90%. Meanwhile, compounds like 2,2′,4,4′-tetrahydroxybenzophenone; 3,4,5,2′,3′,4′-hexahydroxybenzophenone; 4,4′-dihydroxybenzophenone; 2,3,4-trihydroxybenzophenone; and 2,4-dihydroxybenzophenone showed good to moderate XOI activity, with IC_50_ values of 47.59, 69.40, 82.94, 112, and 184 µM, respectively [63,64,65]. Essential oil containing 91.97% (+)-ar-turmerone showed strong antioxidant activity with an IC_50_ value of 35.47 µg/mL. Meanwhile, (+)-ar-turmerone from *Curcuma longa* exhibited XOI activity, achieving 96.01% water-soluble tetrazolium 1 (WST-1) reduction compared to the control, although its effectiveness is still lower than superoxide dismutase (SOD) [66,67]. One of the 4-methoxycinnamic acid compounds, isoferulic acid, exhibited antioxidant activity with an IC_50_ value of 4.58 µg/mL, while its derivative, trans-4-methoxycinnamic acid, showed good xanthine oxidase inhibitory activity with an IC_50_ value of 26.35 μM [68,69]. Ricinine, an alkaloid compound, presented strong antioxidant activity with an IC_50_ value of 63.80 µg/mL [70]. Although no specific reports confirm the XOI activity of ricinine, one study indicated that alkaloid extracts showed XOI activity with an IC_50_ value of 76.33 ppm [71].

## 4. Materials and Methods

### 4.1. General Experiment Procedure

The experimental setup included the following components: silica gel 60 H 5–40 μm (Merck^®^, Darmstadt, Germany), silica gel 60 02–05 mm (Merck^®^, Darmstadt, Germany), silica gel 60 0.063–0.200 mm (Merck^®^, Darmstadt, Germany), and TLC silica gel 60 GF254 (Merck^®^, Darmstadt, Germany); 96-well plates (Corning Inc., Corning, NY, USA); rotavapor: RV 10 (IKA, Staufen, Germany); UV-visible spectrophotometer: Carry 60 UV-Vis (Agilent Technologies, Santa Clara, CA, USA); Microplate reader: Multiskan Skyhigh (Thermo Scientific, Waltham, MA, USA); LC-MS-MS: Thermo Scientific™ Vanquish™ UHPLC Binary Pump (Thermo Scientific, Waltham, MA, USA) and Thermo Scientific™ Q Exactive™ Hybrid Quadrupole-Orbitrap™ High-Resolution Mass Spectrometer ((Thermo Scientific, Waltham, MA, USA). For testing total phenol and flavonoid content, the main reagents were gallic acid (Sigma-Aldrich, St. Louis, MO, USA), quercetin (Sigma-Aldrich, St. Louis, MO, USA), Folin-Ciocalteu reagent (Sigma-Aldrich, St. Louis, MO, USA), and AlCl_3_ (Merck^®^, Darmstadt, Germany). In assessing antioxidant activity, the primary reagents were 2,4,6-tris(2-pyridyl)-s-triazine (TPTZ) (Sigma-Aldrich, St. Louis, MO, USA), 2,2-diphenyl-1-picrylhydrazyl (DPPH) (Sigma-Aldrich, St. Louis, MO, USA), ascorbic acid (Sigma-Aldrich, St. Louis, MO, USA), and neocuproine (Sigma-Aldrich, St. Louis, MO, USA). For xanthine oxidase inhibitory activity, the key reagents were xanthine oxidase (Sigma-Aldrich, St. Louis, MO, USA) and xanthine (Sigma-Aldrich, St. Louis, MO, USA).

### 4.2. Material

The purple-orange variety of sweet potato, characterized by its purple skin and orange flesh, was collected from a plantation in Tasikmalaya Regency, West Java, Indonesia, in June 2023. The species identification was performed at the Department of Biology, Faculty of Mathematics and Natural Sciences, Padjadjaran University (No.43/HB/03/2023).

### 4.3. Preparation of Crude Drugs and Extraction

Preparation includes gathering fresh plant material, wet sorting, washing, slicing, drying, dry sorting, and grinding into a dry powdered crude drug. The dried crude drug powder was then sieved to obtain particles around 3.28 mm in size. The first extraction phase targets the purple-orange sweet potato variety’s tubers, stems, and leaves. Each part of the plant, 300 g of powdered crude drug, was extracted using reflux with 96% ethanol as the solvent for 2.5 h after reaching boiling point. The reflux process was repeated three times. The liquid was then reduced in volume using a rotavapor to obtain thick extracts from each plant part: ethanol leaves extract (ELE), ethanol stem extract (ESE), and ethanol tuber extract (ETE). The selection of 96% ethanol and the reflux method was based on prior research findings, particularly regarding total phenolic and flavonoid contents that influence antioxidant and xanthine oxidase inhibitory activities [6,7,8,14,72,73,74].

In the second phase of extraction, the target was the leaves. Powdered crude drug (300 g) was extracted by reflux for 2.5 h after reaching boiling point. First, n-hexane was used for extraction (three times), followed by ethyl acetate for the residue (three times), and finally, ethanol for the residue (three times). After concentration, three extracts were obtained: n-hexane leaves extract (HE), ethyl acetate leaves extract (EAE), and ethanol leaves extract (EE) [8].

### 4.4. Fractionation Using Vacuum Liquid Chromatography (VLC)

Fractionation was performed to separate the compounds in the extract based on polarity, producing combined fractions that simplify the next steps. Fractionation was performed using silica gel 60 H (5–40 μm) as the stationary phase, with gradient elution of a chloroform–ethyl acetate combination (ranging from 9:1 to 1:9) as the mobile phase, which was selected based on prior TLC monitoring of the ethyl acetate extract. Thin-layer chromatography was applied to monitor the fractions.

### 4.5. Subfractionation Using Classic Column Chromatography (CCC)

Silica gel 60 (0.063–0.200 mm) was used as the stationary phase and isocratic elution with chloroform–ethyl acetate (7:3) as the mobile phase, monitored by thin-layer chromatography.

### 4.6. Thin Layer Chromatography (TLC) Procedure

Samples were applied at the bottom edge. Plates were placed in a saturated chamber with the mobile phase, allowing it to flow to the specified upper edge. The separation outcomes were examined under UV light λ 366 and 254 nm after spraying with sulfuric acid, FeCl_3_, citroborate, DPPH (2,2-diphenyl-1-picrylhydrazyl), and CUPRAC (Cupric Reducing Antioxidant Capacity).

### 4.7. Estimation of Total Phenol Content (TPC)

The modified Pourmorad method [75] was employed to determine the total phenolic content (TPC). A 50 μL aliquot of each sample was combined with 500 μL of 10% Folin-Ciocalteu reagent and 400 μL of 1 M NaOH. The solution was incubated for 30 min, and absorbance was evaluated at λ 765 nm. The findings were presented as milligrams of gallic acid equivalents per gram of sample (mg GAE/g), which was applied to the extract similarly using a calibration curve of gallic acid (60 to 130 μg/mL).

### 4.8. Quantification of Total Flavonoid Content (TFC)

The total flavonoid content (TFC) was measured using the modified Chang method [76]. Each sample (100 μL) was mixed with 20 μL 1 M sodium acetate, 20 μL 10% AlCl_3_, 300 μL methanol, and 560 μL distilled water. Thirty minutes were spent incubating the mixtures, and absorbance was determined at λ 415 nm. The findings were revealed as milligrams of quercetin equivalents per gram of sample (mg QE/g sample), using a quercetin standard calibration curve ranging from 40 to 110 μg/mL, similarly applied to the sample. The AlCl_3_ colorimetric method was selected due to its superior stability, reproducibility, and recovery.

### 4.9. DPPH (2,2-Diphenyl-1-picrylhydrazyl) Assay

The testing method followed the procedure of previous research with some modifications [77]. Between 10 and 40 μL of ascorbic acid (200 μg/mL) was added to an Eppendorf tube, followed by methanol to reach a final volume of 125 μL. Afterward, 750 μL of DPPH solution (50 μg/mL) was gained, the mixture was left to react for 30 min, and absorbance was recorded at 517 nm. The percent inhibition for each concentration was calculated, and a calibration curve was created to derive the ascorbic acid regression equation.

The sample testing followed the same steps as the ascorbic acid test, using 12.5 μL of the sample (10,000 μg/mL). Sample antioxidant activity was determined by inputting the sample percent inhibition into the ascorbic acid regression equation. Antioxidant activity was indicated as ascorbic acid equivalents, i.e., mg ascorbic acid equivalent antioxidant capacity (AEAC) per gram of sample.

### 4.10. FRAP (Ferric Reducing Antioxidant Power) Assay

The testing method followed the procedure of previous research with some modifications [78]. A total of 20 mg of ascorbic acid was weighed and dissolved in 100 mL of methanol. Various concentrations were prepared: 120 μL, 100 μL, 80 μL, 60 μL, 40 μL, and 20 μL. Each concentration was diluted with distilled water to 500 µL, then 500 µL of FRAP solution was mixed in. The mixtures were left for 30 min, and absorbance was recorded at a wavelength of 595 nm. The percentage increase in capacity for each concentration was calculated, a calibration curve for the increase in ascorbic acid capacity was created, and the regression equation was obtained.

The sample testing procedure followed the same method as ascorbic acid, using 50 μL of the sample (10,000 μg/mL). The percentage increase in capacity for the sample was determined. The antioxidant activity of the sample was calculated by inputting the sample’s percent increase in capacity into the ascorbic acid regression equation. Antioxidant activity was denoted as ascorbic acid equivalents, mg AEAC per gram of sample.

### 4.11. CUPRAC (Cupric Ion Reducing Antioxidant Capacity)

The testing method was modified from previous research [78]. An Eppendorf tube was prepared with 15–35 μL of ascorbic acid (200 μg/mL) and ammonium acetate buffer to reach a final volume of 250 μL. Then, 750 μL of CUPRAC solution (100 μg/mL) was gained, the mixtures were left for 30 min, and absorbance was recorded at 450 nm. The percentage increase in capacity for each concentration was calculated, a calibration curve was generated for the increase in ascorbic acid capacity, and the regression equation was obtained.

The procedure for testing samples followed the same method as for ascorbic acid, using 12.5 μL of the sample (10,000 μg/mL). The percentage increase in capacity for the sample was measured. The antioxidant capacity of the sample was determined using the percentage increase based on the ascorbic acid regression equation. The antioxidant activity was reported as ascorbic acid equivalents, mg AEAC per gram of sample.

### 4.12. Xanthine Oxidase Inhibitory (XOI) Activity

The XOI activity was assessed using 96-well Corning plates and a microplate reader, following the methods of Noro, Owen, and Duong with slight adjustments [79,80,81]. To prepare a 100 μg/mL solution of allopurinol, 1 mg of allopurinol was dissolved in 200 µL DMSO, and phosphate buffer (pH 7.5) was added to achieve a final volume of 1000 µL. Serial dilutions were performed to achieve 16–40 μg/mL concentrations. The mixture was prepared by combining 50 µL of allopurinol from each concentration series, 50 µL of phosphate buffer (pH 7.5), and 50 µL of enzyme solution (0.15 unit/mL xanthine oxidase in phosphate buffer pH 7.5), then preincubating for 15 min at 24 °C. The process was started by introducing 50 µL of substrate solution (0.15 mM xanthine in phosphate buffer pH 7.5). The test solution was left to react at 24 °C for 30 min. A blank was prepared similarly, but without the allopurinol, and absorbance was investigated at λ290 nm. The analysis for allopurinol was performed in six repetitions. The inhibitory activity of xanthine oxidase was determined using the formula: I% = [(A − B)/A] × 100, where A = absorbance of enzyme xanthine oxidase without the test allopurinol minus the blank of A (absorbance without xanthine oxidase and allopurinol), and B = absorbance of the allopurinol minus the blank of B (absorbance without xanthine oxidase). The percent inhibition for each concentration was calculated, and a calibration curve was created to derive the allopurinol regression equation.

The sample testing procedure followed the same method as allopurinol, using 50 μL of the sample (400–1000 μg/mL). Sample xanthine oxidase inhibitory activity was determined by inputting the sample percent inhibition into the allopurinol regression equation. Xanthine oxidase inhibitor activity was expressed as allopurinol equivalent, i.e., mg allopurinol equivalent xanthine oxidase inhibitory capacity (AEXIC) per gram of sample.

### 4.13. Identification LC-MS-MS

A Thermo Scientific™ Accucore™ Phenyl-Hexyl column (Thermo Scientific, Waltham, MA, USA) (100 mm × 2.1 mm, 2.6 µm) at 40 °C was used with a mobile phase of MS-grade water (0.1% formic acid) and methanol (0.1% formic acid). Gradient elution progressed from 5% to 90% B over 16 min, held for 4 min, and returned to 5% by 25 min at a 0.3 mL/min flow rate. The sample (1 mg/mL in 70% methanol) had a 3 µL injection volume. ESI positive mode was applied with a 3.30 kV capillary voltage and 320 °C temperature, scanning 66.7–1000 *m*/*z*. Compounds were identified using the MzCloud, ChemSpider, and PubChem databases.

### 4.14. Statistical Analysis

All data were analyzed using SPSS version 21. Parametric analysis used One-Way ANOVA and Tukey’s post hoc test. Meanwhile, non-parametric analysis used the Kruskal–Wallis and Mann–Whitney post hoc tests.

## 5. Conclusions

The extract, fractions, and subfractions of purple-orange sweet potato leaves exhibited antioxidant and xanthine oxidase inhibitory activities. A noticeable trend of decreased activity, particularly in antioxidant activity, was observed from the extracts to the fractions and subfractions. In terms of activity potential, the extracts appear more promising for development into herbal product raw materials compared to the fractions or subfractions. However, the safety profiles may differ, so toxicity testing of the extract, fractions, and subfractions is necessary.

## Figures and Tables

**Table 1:** Determination of total flavonoids, total phenols, inhibitory activity of xanthine oxidase, and antioxidant activities of extracts from purple-orange sweet potato plant parts.

Ethanol Extract	Yield (%)	Total Phenols (mg GAE/g)	Total Flavonoids (mg QE/g)	DPPH (mg AEAC/g)	FRAP (mg AEAC/g)	CUPRAC (mg AEAC/g)	XOI Activity (mg AEXIC/g)
Leaves	24.120	131.187 ± 0.110 ^a^	149.959 ± 0.277 ^a^	333.047 ± 28.172 ^a^	452.609 ± 0.112 ^a^	982.728 ± 3.492 ^a^	69.131 ± 1.400 ^a^
Stems	19.960	27.184 ± 1.740 ^b^	15.469 ± 0.011 ^b^	21.117 ± 3.440 ^b^	23.395 ± 0.045 ^b^	92.120 ± 1.426 ^b^	8.329 ± 2.741 ^b^
Tubers	14.430	12.347 ± 0.027 ^c^	22.062 ± 0.118 ^c^	6.979 ± 2.905 ^c^	9.782 ± 0.079 ^c^	42.232 ± 0.889 ^c^	1.121 ± 0.729 ^c^

AEAC = ascorbic acid equivalent antioxidant capacity; AEXIC = allopurinol equivalent xanthine inhibitory capacity; QE = quercetin equivalent; GAE = gallic acid equivalent. a–c: distinct letters in one column exhibit significant differences (*p* < 0.05); *n* = 6.

**Table 2:** Determination of total flavonoids, total phenols, inhibitory activity of xanthine oxidase, and antioxidant activities of purple-orange sweet potato leaf extract.

Extract	Yield (%)	Total Phenols (mg GAE/g)	Total Flavonoids (mg QE/g)	DPPH (mg AEAC/g)	FRAP (mg AEAC/g)	CUPRAC (mg AEAC/g)	XOI Activity (mg AEXIC/g)
n-Hexane	4.233	90.697 ± 0.373 ^a^	161.153 ± 0.445 ^a^	138.181 ± 0.094 ^a^	52.267 ± 0.607 ^a^	298.181 ± 1.221 ^a^	4.417 ± 2.393 ^a^
Ethyl acetate	10.478	142.776 ± 0.083 ^b^	76.591 ± 0.264 ^b^	511.212 ± 0.416 ^b^	90.837 ± 0.149 ^b^	237.944 ± 1.711 ^b^	45.192 ± 4.981 ^b^
Ethanol	6.787	182.803 ± 0.057 ^c^	65.786 ± 0.336 ^c^	1067.407 ± 4.173 ^c^	161.179 ± 0.268 ^c^	409.556 ± 1.623 ^c^	15.870 ± 6.523 ^c^

AEAC = ascorbic acid equivalent antioxidant capacity; AEXIC = allopurinol equivalent xanthine inhibitory capacity; QE = quercetin equivalent; GAE = gallic acid equivalent. a–c = distinct letters in one column exhibit significant differences (*p* < 0.05); *n* = 6.

**Table 3:** Pearson correlation analysis of the purple-orange sweet potato leaves extract.

	Pearson Correlation Coefficient (r)			
	DPPH	FRAP	CUPRAC	XOI
Total phenols	0.981 ^a^	0.970 ^a^	0.580 ^b^	0.339 ^c^
Total flavonoids	−0.861	−0.833	−0.270	−0.618

a = perfect correlation, b = moderate correlation, and c = weak correlation.

**Table 4:** Total flavonoids, total phenols, inhibitory activity of xanthine oxidase, and antioxidant activities of fractions from the ethyl acetate leaves extract.

Fraction	Yield (%)	Total Phenols (mg GAE/g)	Total Flavonoids (mg QE/g)	DPPH (mg AEAC/g)	FRAP (mg AEAC/g)	CUPRAC (mg AEAC/g)	XOI Activity (mg AEXIC/g)
CF1	11.804	27.965 ± 0.184 ^a^	135.297± 0.222 ^a^	30.844 ± 0.054 ^a^	3.658 ± 0.346 ^a^	222.410± 1.950 ^a^	ND
CF2	8.773	36.242 ± 0.045 ^b^	107.731 ± 0.865 ^b^	145.416 ± 0.190 ^b^	22.910 ± 0.047 ^b^	384.054 ± 4.625 ^b^	6.377 ± 1.677 ^a^
CF3	19.293	109.283 ± 0.613 ^c^	155.343± 0.477 ^c^	82.001 ± 0.150 ^c^	81.144 ± 0.220 ^c^	705.175 ± 3.308 ^c^	ND
CF4	17.233	28.188 ± 0.098 ^d^	48.117 ± 0.111 ^d^	66.914 ± 0.417 ^d^	34.621 ± 0.018 ^d^	ND	ND
CF5	19.005	62.453 ± 0.094 ^e^	56.926 ± 0.255 ^e^	158.475 ± 0.170 ^e^	86.849 ± 0.048 ^e^	1008.892 ± 1.620 ^d^	6.062 ± 1.730 ^a^

AEAC = ascorbic acid equivalent antioxidant capacity; AEXIC = allopurinol equivalent xanthine inhibitory capacity; QE = quercetin equivalent; GAE = gallic acid equivalent. ND = not detected. a–e: distinct letters in one column exhibit significant differences (*p* < 0.05); *n* = 6.

**Table 5:** Determination of total flavonoids, total phenols, inhibitory activity of xanthine oxidase, and antioxidant activity of subfractions from CF5.

Sample	Total Phenols (mg GAE/g)	Total Flavonoids (mg QE/g)	DPPH (mg AEAC/g)	FRAP (mg AEAC/g)	CUPRAC (mg AEAC/g)	XOI Activity (mg AEXIC/g)
CSF1	19.069 ± 0.065 ^a^	36.592 ± 0.185 ^a^	154.143 ± 4.593 ^a^	ND	ND	7.583 ± 1.29 ^a^
CSF2	28.961 ± 0.056 ^b^	51.479 ± 0.032 ^b^	42.502 ± 0.150 ^b^	ND	ND	ND
CSF3	21.942 ± 0.055 ^c^	28.388 ± 0.028 ^c^	51.878 ± 0.242 ^c^	ND	ND	9.620 ± 1.508 ^b^
CSF4	6.980 ± 0.017 ^d^	ND	50.286 ± 0.205 ^c^	ND	ND	25.367 ± 0.559 ^c^
CSF5	11.694 ± 0.01 ^e^	32.603 ± 0.074 ^d^	41.513 ± 0.239 ^b^	ND	ND	ND

AEAC = ascorbic acid equivalent antioxidant capacity; AEXIC = allopurinol equivalent xanthine inhibitory capacity; GAE = gallic acid equivalent; QE = quercetin equivalent; ND = not detected. a–e = distinct letters in one column exhibit significant differences (*p* < 0.05); *n* = 6.

**Table 6:** Comparison of antioxidant and xanthine oxidase inhibitory activity among ELE, EAE, CF5, and CSF3.

Sample	DPPH (mg AEAC/g)	FRAP (mg AEAC/g)	CUPRAC (mg AEAC/g)	XOI Activity (mg AEXIC/g)
ELE	333.047 ± 28.172 ^a^	452.609 ± 0.112 ^a^	982.728 ± 3.492 ^a^	69.131 ± 1.400 ^a^
EAE	511.212 ± 0.416 ^b^	90.837 ± 0.149 ^b^	237.944 ± 1.711 ^b^	45.192 ± 4.981 ^b^
CF5	158.475 ± 0.170 ^c^	86.849 ± 0.048 ^c^	1008.892 ± 1.620 ^c^	6.062 ± 1.730 ^c^
CSF3	51.878 ± 0.242 ^d^	ND	ND	9.620 ± 1.508 ^d^

AEAC = ascorbic acid equivalent antioxidant capacity; AEXIC = allopurinol equivalent xanthine inhibitory capacity; ND = not detected. a–d: distinct letters in one column exhibit significant differences (*p* < 0.05); *n* = 6.

**Table 7:** Tentatively identified compounds of subfraction CSF3 of purple-orange sweet potato by UPLC–MS/MS.

No.	Rt (min.)	Identified Compounds	Molecular Formula	Molecular Weight	Concentration (%)
1	18.419	Cholest-4-en-3-one	C_27_H_44_O	384.338	0.109
2	10.45	4-hydroxy-6-[2-(2-methyl-1,2,4a,5,6,7,8,8a-octahydronaphthalen-1-yl)ethyl]oxan-2-one	C_18_H_28_O_3_	292.204	0.021
3	11.759	4-methylbenzophenone	C_14_H_12_O	196.089	0.014
4	11.063	Benzophenone	C_13_H_10_O	182.073	0.061
5	10.817	Tangeritin	C_20_H_20_O_7_	372.121	0.011
6	12.969	(+)-ar-Turmerone	C_15_H_20_O	216.152	0.017
7	15.066	4-Methoxycinnamic acid	C_10_H_10_O_3_	178.063	0.494
8	4.63	Ricinine	C_8_H_8_N_2_O_2_	164.059	0.010

## Data Availability

Derived data supporting the findings of this study are available from the corresponding author on request.
